# Nasal phaeohyphomycosis in a cat caused by *Cladophialophora boppii*

**DOI:** 10.1093/jvimsj/aalag235

**Published:** 2026-10-03

**Authors:** Julia McCarthy, Monica Augusto, Emma O'Neill, Ignacio Otero Balda, Eleanor Wyatt

**Affiliations:** School of Veterinary Medicine, University College Dublin, Dublin D04 W6F6, Ireland; School of Veterinary Medicine, University College Dublin, Dublin D04 W6F6, Ireland; School of Veterinary Medicine, University College Dublin, Dublin D04 W6F6, Ireland; School of Veterinary Medicine, University College Dublin, Dublin D04 W6F6, Ireland; Northwest Veterinary Specialists, Part of Linnaeus Ltd, Runcorn WA7 3FW, United Kingdom

**Keywords:** cutaneous, feline, fungal, itraconazole, nasal planectomy, pigmented

## Abstract

Phaeohyphomycosis is an opportunistic mycosis caused by dematiaceous fungi, typically affecting the cutaneous or subcutaneous tissues of the facial region and distal extremities in cats. This report documents a case *of Cladophialophora boppii* infection in a cat. A 7-year-old neutered male domestic shorthair cat was presented with a chronic, pigmented nasal lesion, non-productive sneezing, and stertor. Computed tomography, rhinoscopy, and tissue sampling were performed. Fungal culture and molecular sequencing confirmed the diagnosis. Oral itraconazole treatment failed to arrest progression, necessitating delayed nasal planectomy. Despite transient remission, disease recurred, requiring repeat surgical debridement, and prolonged antifungal treatment. Clinical resolution was ultimately achieved, with marked improvement in the cat’s quality of life. Identification of a novel species of phaeohyphomycosis in a cat emphasizes the need for increased awareness of emerging fungal pathogens that can precipitate considerable morbidity, particularly in the absence of prompt aggressive intervention.

## Introduction

Phaeohyphomycoses refers to a diverse group of infections caused by darkly pigmented (melanized) filamentous fungi proliferating within host tissues.^1^ Numerous genera have been implicated, most commonly *Alternaria, Cladophialophora, Microsphaeropsis, Lomento-spora*, and *Exophiala* species.^2^ This class of saprophytic organisms is ubiquitous in the environment and has a worldwide distribution. Although disease remains uncommon, the incidence of infection continues to increase in humans and animals.^2–6^ Infections are typically opportunistic and occur as a result of traumatic inoculation from the environment (soil, wood, and decomposed plants). Predilection sites include the facial region (nose) and distal extremities (digits) because they are most frequently in contact with the surrounding environment.^1^ The distribution is usually focal or multifocal local invasion within cutaneous or subcutaneous tissues, but disseminated disease has been described.^1^ Both immunocompetent and immunocompromised cats may be affected, with immunosuppression recognized as a risk factor for disease development and hematogenous spread.^1^^,^^2^

Phaeohyphomycosis typically can be distinguished from other dermatopathies by the presence of lesional pigmentation.^1^ Their melanin pigment is a key virulence factor, and systemic infections in humans often are associated with a poor prognosis.^7^ Diagnosis is achieved by visualization of melanized fungal elements on cytology or histopathology. Special stains such as Fontana-Masson, Grocott-Gomori’s methenamine silver or periodic acid-Schiff, highlight fungal cell walls and melanin, thus optimizing diagnostic sensitivity.^2^ Sabouraud’s dextrose agar (SDA), malt extract agar, inhibitory mold agar and potato dextrose agar, support growth of phaeohyphomycosis, at an optimal temperature of 25-37 °C.^2^ Identification of phaeohyphomycosis genera and species necessitates molecular testing by panfungal polymerase chain reaction (PCR), preferentially of DNA extracted from fungal culture of diseased tissue, but DNA obtained directly from formalin-fixed paraffin-embedded or fresh or frozen tissue samples also can be used.^2^ Sequencing of DNA then can be utilized for species confirmation.^2^

*Cladophialophora boppii* has been described rarely in humans and to our knowledge not in animals. Previous case reports have detailed cutaneous, nail, ocular, and pulmonary transplant tissue infections.^8–11^ Thus far, only *Cladophialophora bantiana* and one case of *Cladophialophora carrionii* have been documented as pathogenic in cats.^12^^,^^13^  *C. bantiana* is predominantly neurotropic, classically causing central nervous system disease in cats, dogs, and humans.^12^^,^^14^ However, nasal cutaneous, systemic, pulmonary, and renal disease also have occurred in cats with *C. bantiana* infection.^15–18^ Similarly, subcutaneous and systemic disease has been reported in dogs.^19^^,^^20^ The single *C. carrionii* case in a cat documented fungal peritoneal effusion.^13^

The identification of a novel species of phaeohyphomycosis (*C. boppii*) in an animal adds to the growing number of reported cases of invasive mycoses, which may be influenced by environmental change.^2–6^

## Case description

A 7-year-old, 5.6 kg, male neutered domestic shorthair cat was presented with a 4-month history of chronic nonproductive sneezing, stertorous respiratory noises, with dark pigmentation and intermittent ulceration of the dorsal rhinarium. The cat lived in a single cat household, had no travel history, and had access to indoors and outdoors. The severity of the clinical signs had fluctuated over time with a poor response to symptomatic treatment. Concomitant health issues included congenital convergent strabismus. The cat displayed moderate to marked stertorous breathing with referred upper respiratory tract noise on thoracic auscultation. Nasal assessment identified mild protuberance and dark pigmentation of the rostral dorsal rhinarium without ulceration, necrosis, exudation, or nodularity (Figure 1A). Mild bilateral submandibular lymphadenomegaly was noted, but the remaining physical examination was unremarkable.

**Figure 1:**
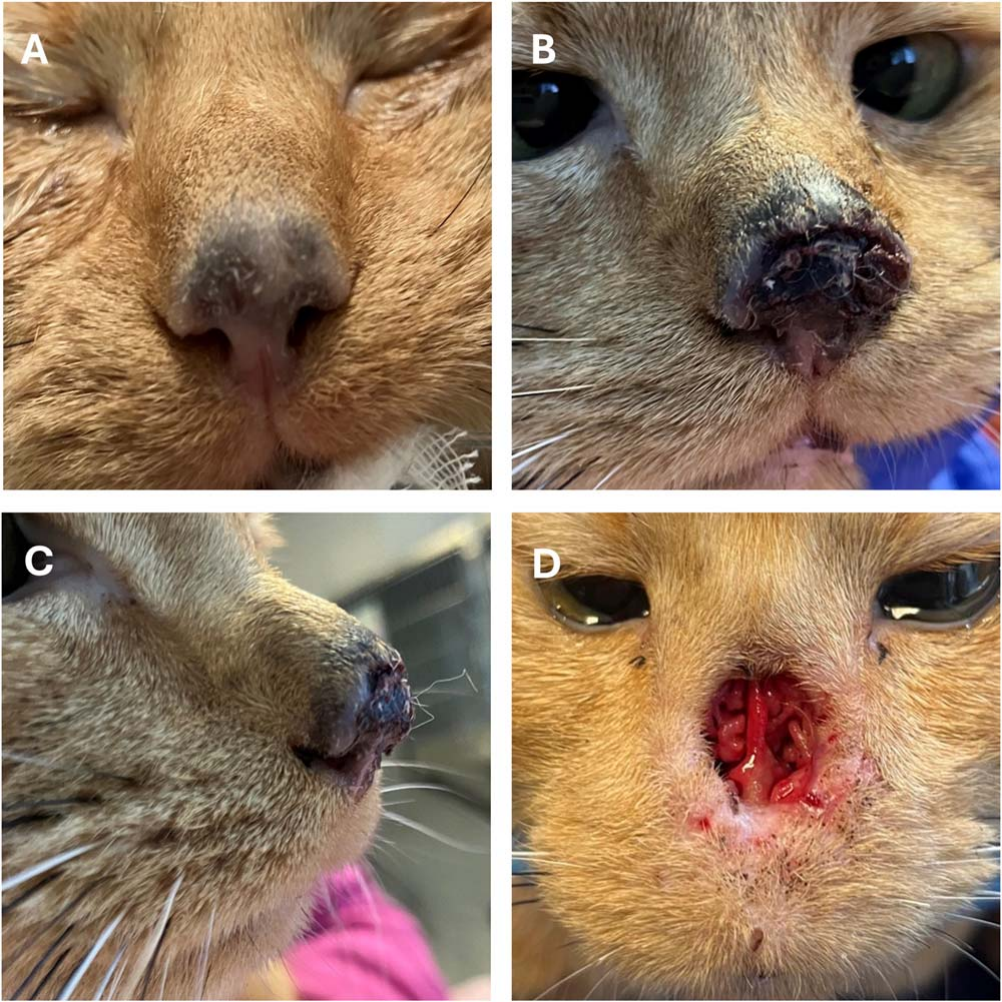
Progression of nasal phaeohyphomycosis lesion. (A) Pigmented nasal lesion at initial presentation. (B, C) Nasal lesion fifteen weeks post initial presentation and instigation of itraconazole treatment. Substantial necrosis, of the most distal, dorsal aspect of the nose is noted. (D) Healed surgical site after the second surgery.

Hematology and biochemistry identified no clinically relevant abnormalities. Serology for feline immunodeficiency virus antibody and feline leukemia virus antigen (IDEXX-SNAP FIV/FeLV Combo Test) was negative.

On contrast-enhanced CT the left nostril and a portion of the right were completely obliterated by mildly contrast enhancing, soft tissue attenuating material, which extended rostro-caudally into both nasal cavities (Figure 2A-C). The lesion caused dorsal rhinarium deformity with bilateral nasal bone erosion and mild mandibular lymph node enlargement. No cervical or thoracic abnormalities were identified. Abdominal imaging was not performed.

**Figure 2:**
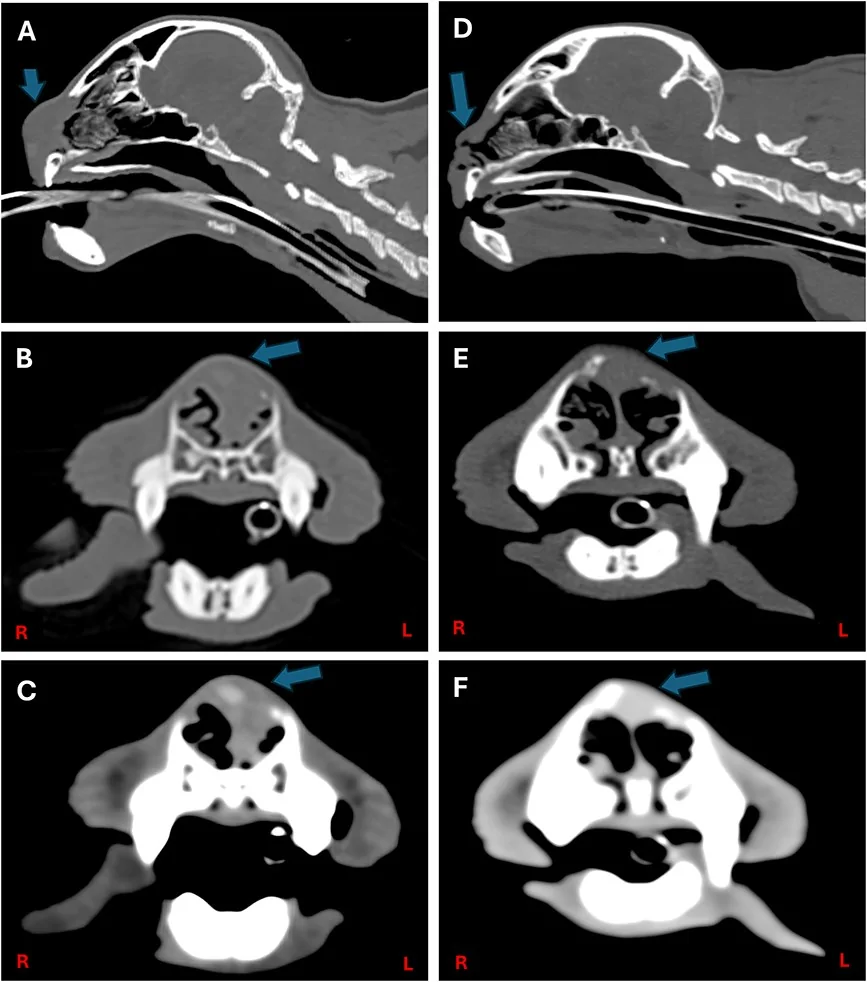
Contrast-enhanced computed tomography of the phaeohyphomycosis-associated nasal lesion (Arrows), shown in sagittal bone view and transverse bone and soft tissue views. (A-C) At initial presentation. (D-F) Twenty-three weeks post initial presentation. The left nostril and a portion of the right were completely obliterated by mildly contrast-enhancing, soft tissue attenuating material. The lesion created a moderate convex deformity of the dorsal aspect of the nose that extended rostro-caudally into both nasal cavities, with the left side being more severely affected.

Retrograde rhinoscopy was unremarkable. Antegrade rhinoscopy was precluded by the cat’s size. Cytology of a fine-needle aspirate from the nasal lesion identified fungal hyphae and yeast-like elements with marked, mixed, neutrophilic (non-septic), and macrophagic inflammation (Supplementary file; Figure 3). Three punch biopsy samples were obtained from the lesion, one of which was submitted for histopathologic evaluation. Examination indicated severe granulomatous dermatitis with intralesional pigmented fungal yeast and hyphal forms, consistent with cutaneous phaeohyphomycosis (Supplementary material; Figure 4).

**Figure 3:**
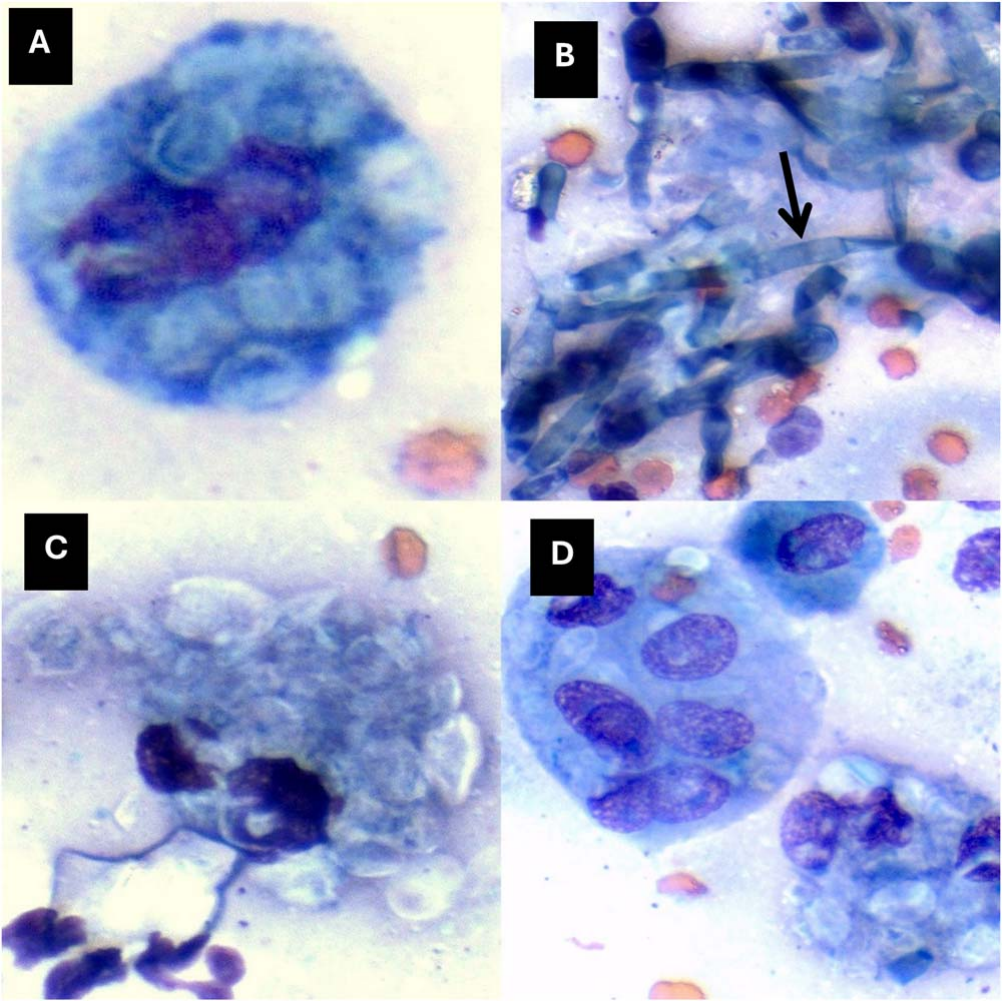
Cytology of fine needle aspirates from the phaeohyphomycosis nasal lesion demonstrating fungal hyphae (black arrow) and yeast-like elements. A marked mixed inflammatory response, consisting of neutrophils (non-septic) and macrophages, is also present. Wright-Giemsa; 10× ocular lens magnification. Total ocular lens and digital magnification (A): 1300×, (B) 654×, (C) 778×, (D) 654×.

**Figure 4:**
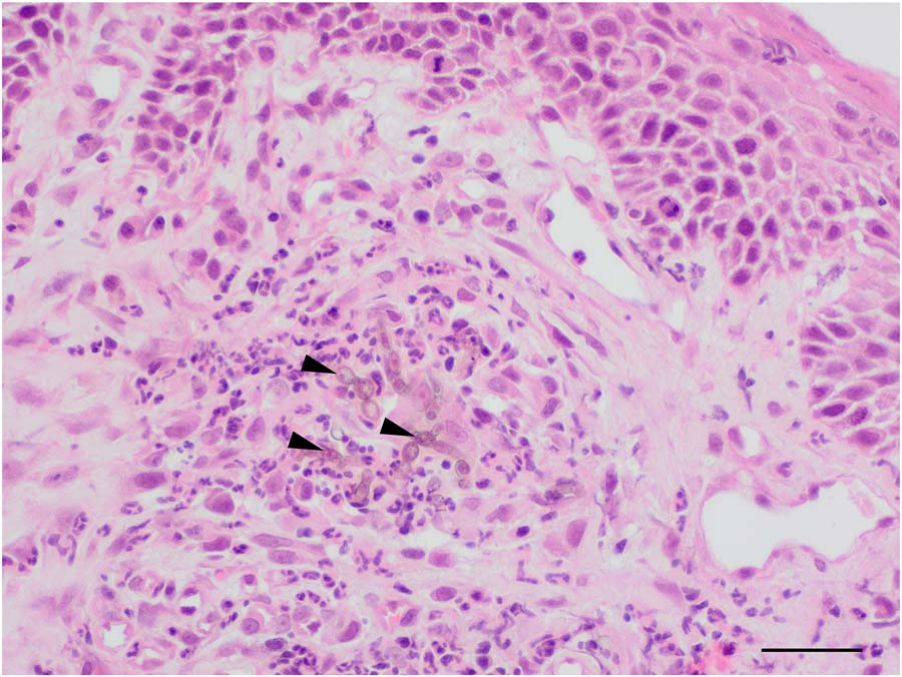
Histopathology of a biopsy sample from the phaeohyphomycosis nasal lesion. Haired skin with dermal infiltration of numerous pigmented fungal yeast and hyphae (arrowheads) surrounded by neutrophils and macrophages. Hematoxylin and eosin. Size bar: 25 μm.

A second biopsy sample was cultured on Sabouraud's dextrose agar (SDA; Oxoid, Thermo Fischer Scientific, Basingstoke, United Kingdom) at the university microbiology laboratory, isolating a slow growing, darkly pigmented fungus after 3 weeks at 25 °C (Figure 5)*.* The third biopsy sample was frozen in phosphate-buffered saline at –80 °C and submitted to the Mycology Reference Centre Manchester (MRCM) for culture and molecular identification. Sequencing was provided by Eurofins Genomics GmbH, Germany. Amplification by PCR and sequencing of the internal transcribed spacers (ITS) and β-tubulin region (using ITS5/4) followed by comparison to reference sequences confirmed the organism as *C. boppii*  ^21–23^ (Supplementary material). Definitive speciation results became available ten weeks after the cat’s initial presentation. Antifungal susceptibility testing was performed at MRCM, according to the EUCAST guidelines. Testing indicated a minimum inhibitory concentration (MIC) > 8 μg/mL for itraconazole and all other antifungals. Interpretive breakpoints for this species have not been established, and the clinical relevance of these results could not be determined.

**Figure 5:**
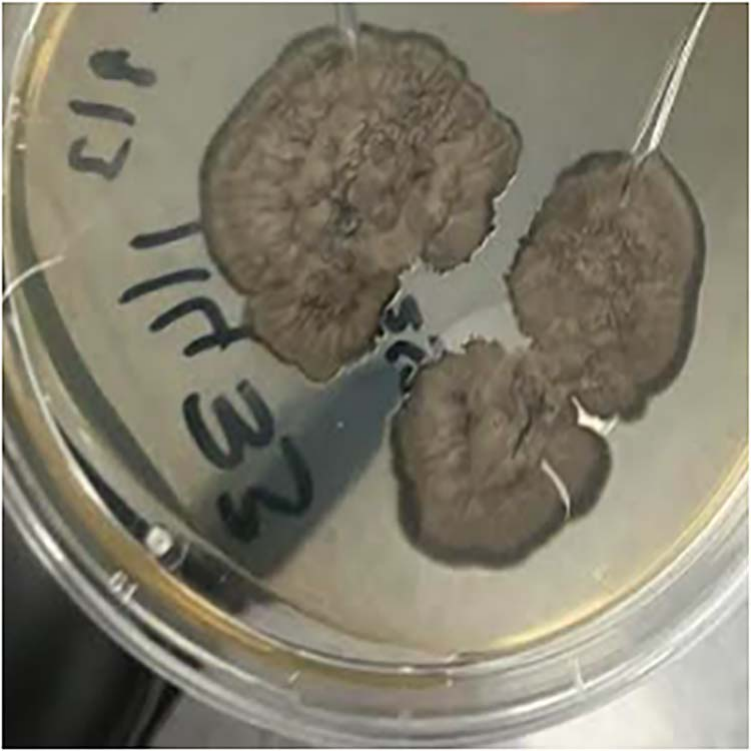
The slow growing, darkly pigmented fungus isolated on Sabouraud dextrose agar at the university microbiology laboratory after 3 weeks of culture at 25 °C.

Oral itraconazole (Intrafungol 10 mg/mL, Lusomedicamenta, Sociedade Técnica Farmacêutica, S.A., Barcarena, Portugal; 5 mg/kg PO q12h) was empirically started based on the histopathologic diagnosis of phaeohyphomycosis, while pending species identification and susceptibility testing. Surgical debridement initially was declined by the owners. At 7 weeks, clinical signs remained static, but by week 15, the lesion had progressed substantially (Figure 1B and C). The cat developed severe necrosis of the nasal planum, pronounced stertor, anorexia, and marked weight loss (approximately 2 kg; body condition score [BCS], 2/9). Clinical examination identified jaundice, dry mucous membranes, prolonged capillary refill time, weak pulses, moderate tachycardia (200 bpm), and enophthalmos. Hematology was unremarkable. Biochemistry identified mild hyperbilirubinemia (1.87 mg/dL; reference interval [RI], 0.1-0.3) and marked increases in alkaline phosphatase (433.8 U/L; RI, 0-66), alanine aminotransferase (381 U/L; RI, 0-45), and aspartate transferase (142 U/L; RI, 0-70) activities. Further diagnostics, including abdominal ultrasonography and a bile acid stimulation test, were not pursued because the results were unlikely to alter the treatment plan given the advanced stage of disease.

The cat was hospitalized and treated with compounded sodium lactate IV fluid therapy (Aqupharm 11, Infomed Fluids SRL, Bucharest, Romania; 5 mL/kg/h), buprenorphine (Buprecare Multidose .3 mg/mL, Produlab Pharma B.V., Raamsdonksveer, the Netherlands; 20 μg/kg IV q8h), maropitant (Vominil 10 mg/mL, VetViva Richter GmbH, Wels, Austria; 1 mg/kg IV q24h), and topical mirtazapine (Mirtaz 20 mg/g, Genera Inc, Rakov Potok, Croatia; 3.8 cm applied to the inner pinna q24h). Because of increased hepatobiliary enzyme activity and hyperbilirubinemia, itraconazole was discontinued. The owner subsequently applied an unspecified formulation and dose of topical terbinafine to the nasal lesion for 2 weeks. During this period, the cat remained hyporexic despite continued topical mirtazapine administration. Terbinafine was discontinued after spontaneous sloughing of the most necrotic aspect of the lesion. Twenty-three weeks after initial presentation, the cat was re-evaluated. Despite marked systemic improvement, the localized nasal lesion remained clinically important. The cat exhibited marked inspiratory dyspnea because of collapse of the nasal planum on inhalation, secondary to destruction of the underlying bony structures (Video 1). At this point, the owner elected to pursue surgical intervention and biochemistry showed resolution of previous hepatobiliary abnormalities.

A second CT of the head, neck, and thorax was performed to assess disease extent and guide surgical planning (Figure 2D-F). Intraoperatively, the infection demonstrated deep tissue invasion, with necrosis of the nasal bone and turbinates. Given the severity of involvement, radical surgical debridement with nasal planectomy was performed instead of a more conservative procedure. Post-operative recovery was uneventful.

Three weeks postoperatively, the surgical site was healed, and the cat had regained normal appetite and weight (4.5 kg; BCS, 4/9). The cat remained disease-free for 5 months before recurrence of phaeohyphomycosis as isolated small brown necrotic plaques on the dorsolateral aspect of the nasal reconstruction. The lesions were surgically debrided and PO itraconazole was reintroduced at a lower dosage (5 mg/kg PO q24h; Figure 1D). Biochemistry for therapeutic monitoring was performed at the primary practice every 2 months and no increase in hepatobiliary enzyme activity was noted.

Ultimately, the fungal infection and associated respiratory signs resolved. Antifungal treatment was provided for 6 months to prevent disease recrudescence after repeat surgical debridement. Despite a complex and protracted clinical course, the outcome was favorable, with substantial improvement in the cat’s quality of life. The cat was alive with no disease recurrence 2 years after initial presentation.

## Discussion

To our knowledge, this case represents the first report of *C. boppii* infection in a cat or any other veterinary species. It contributes to emerging evidence of increasing incidence and geographic spread of invasive fungal infections, a pattern that may be influenced by climate change.^2–6^

Prominent dark nasal pigmentation was an unusual and defining feature of this case. Differential diagnoses included other pigmented fungal infections, such as *Aspergillus niger,*^24^ whereas nasal melanoma was considered less likely, given the absence of underlying dermal pigmentation.^25^

A challenge in this case was the prolonged interval between presentation and molecular identification of the phaeohyphomycosis genus and species, potentially complicating clinical management and delaying targeted treatment. This delay primarily reflected the geographical region, where fungal infections are uncommon, and veterinary resources for fungal molecular testing are limited. This case emphasizes the importance of recognizing phaeohyphomycosis as a differential diagnosis for pigmented nasal lesions to encourage earlier diagnostic and therapeutic intervention in comparable cases. Cytology and histopathology are fundamental to fungal infection diagnosis, particularly when culture and molecular identification are delayed or unavailable. In phaeohyphomycosis, visualization of pigmented fungal elements within pyogranulomatous inflammation on cytology or histology is often sufficient to support a presumptive diagnosis and initiate empirical antifungal treatment, with special stains further enhancing diagnostic sensitivity.^2^ Best practice, however, dictates rapid and accurate species-level identification to enable appropriate antifungal treatment.^26^ In view of the existence of over 100 phaeohyphomycosis species with variable virulence, antifungal susceptibility testing should play a central role in therapeutic planning. Even when susceptibility testing is unavailable, molecular identification supports selection of the most appropriate empirical treatment.^26^ This selection is guided by general information of antifungal susceptibility profiles across different fungal genera and species.^2^^,^^26^ Formal antifungal susceptibility categorization in this case was not possible because of insufficient available data for the *C. boppii* species. This situation is likely to change if as more cases are recognized and data is gathered. Despite this limitation, the reported MIC of > 8 μg/mL for itraconazole exceeded plasma concentrations typically achievable in cats at a dosage of 5 mg/kg, which may have suggested decreased susceptibility.^27^ In this context, surgical intervention likely played a key role in successful management of the infection.

Owner compliance, particularly regarding therapeutic drug monitoring, was an additional limitation in this case. Dose reduction is recommended in cases of itraconazole-associated hepatic injury because lower overall exposure may mitigate hepatotoxicity recurrence in cats.^26^ In this case, although itraconazole-associated hepatotoxicity could not be definitively confirmed, it was considered the primary differential diagnosis for the increased hepatobiliary enzyme activity, which resolved after drug discontinuation, supporting an adverse drug effect. After the second surgical debridement, itraconazole was re-initiated at a decreased dosage of 5 mg/kg once daily, compared with the initial regimen of 5 mg/kg twice daily. An extended 6-month course was provided to prevent disease recrudescence and was well tolerated. Strict therapeutic monitoring accompanied this second course, with serum biochemistry performed every 2 months. Doing so facilitated early detection of potential drug-related adverse effects and ensured treatment adherence, emphasizing the importance of ongoing biochemical monitoring during prolonged antifungal treatment.^26^

Another limitation of this case was the absence of abdominal imaging to screen for comorbidities that may have contributed to immunosuppression or influenced treatment response. Although hematology, biochemistry, and retroviral testing were unremarkable at initial presentation, CT was limited to the head, neck, and thorax, and potential abdominal pathology could not be excluded. This limitation became particularly relevant after the identification of hepatopathy where abdominal imaging may have provided additional insight into underlying hepatic or abdominal lesions affecting immune function, antifungal tolerance, and disease progression.

Current guidelines recommend prolonged antifungal treatment after surgery to minimize the risk of phaeohyphomycosis recurrence.^26^ In this case, extended postoperative antifungal treatment was withheld after the first surgery because of the cat’s history of suspected hepatotoxicity and the aggressive surgical debridement performed. The subsequent disease relapse emphasizes the potential consequences of prematurely discontinuing systemic antifungals, particularly with invasive fungal pathogens such as *C. boppii*.

This case supports the need for early, aggressive, and sustained intervention in phaehyphomycosis infections. Earlier radical surgical excision and appropriate antifungal treatment may have minimized surgical morbidity, disease burden and the need for repeat interventions. This report emphasizes the importance of early accurate diagnosis with fungal species identification, alongside surgical debridement, careful monitoring, and tailored antifungal treatment in managing invasive fungal infections in cats.

Nine case reports have described *C. boppii* causing a spectrum of disease severity in humans. At the milder end, cutaneous and nail disease responded to variable courses of PO terbinafine or itraconazole with adjunctive topical antifungals such as ciclopiroxolamine in onychomycosis management.^8^^,^^9^^,^^28^ A deeper cutaneous infection was successfully managed with surgical excision and 3 weeks of PO itraconazole.^29^ At the severe end, one report detailed ocular disease, and another pulmonary disease in a transplant recipient, both of which were poorly responsive to treatment and associated with substantial morbidity.^10^^,^^11^ The case reported here appears to represent an intermediate phenotype within this spectrum. Unlike the severe cases, disease was ultimately managed with 2 surgical debridements followed by extended itraconazole treatment. The impact of topical terbinafine could not be determined and may represent a potential confounding factor. Because the formulation and dosage were unknown, its clinical effect could not be evaluated. The sloughing of the necrotic nasal planum was more likely attributable to progressive ischemic tissue necrosis rather than a treatment response.

Documented cases of invasive fungal diseases have increased.^2–6^ This situation is likely multifactorial, potentially reflecting both increases in geographic distribution and a growing population of at-risk individuals. The expanding geographical distribution of pathogenic fungi is thought to result from climate change, through rising temperatures, fluctuating precipitation trends, and ecological instability.^4^ Immunocompromised individuals including those receiving organ transplantation, chemotherapy, invasive medical interventions, and patients who have received immunomodulation for COVID-19, are particularly susceptible.^3–6^ Although effective antifungal drugs are currently available, increasing antifungal resistance and relatively limited medical and veterinary research into mycology and antifungal treatment remain important concerns.^26^

In summary, our report documents a case of *C. boppii* infection in a cat and provides insight into the management of this emerging pathogen. Phaeohyphomycosis should be considered a differential diagnosis for pigmented nasal and cutaneous lesions in cats, regardless of immunologic status. Successful outcomes necessitate early, aggressive surgical debridement alongside prolonged, carefully monitored antifungal treatment. Given that treatment delays or interruptions frequently lead to disease progression or recurrence, clinical vigilance is essential in addressing the substantial morbidity associated with these fungal infections.

## Supplementary Material

Supplementary_material_aalag235

## Off-label antimicrobial declaration

Authors declare no off-label use of antimicrobials.

## Institutional animal care and use committee or other approval declaration

Informed consent (verbal and written) was obtained from the owner of the animal described in this work for all treatment and procedures undertaken.

## Abbreviations

BCS: body condition score
bpm: beats per minute
CT: computed tomography
MIC: minimum inhibitory concentration
MRCM: Mycology Reference Centre Manchester
RI: reference interval
SDA: Sabouraud’s dextrose agar
