# Supplementary material for: Nasal phaeohyphomycosis in a cat caused by Cladophialophora boppii
Source: J Vet Intern Med. 2026 Oct 3;40(5):aalag235. doi: 10.1093/jvimsj/aalag235 (PMC13633654; doi:10.1093/jvimsj/aalag235)
Supplement: Supplementary file 1 [file supplementary_material_aalag235.zip › Supplementary_caption.docx]

VIDEO 1: At twenty-three weeks after initial presentation, the cat had marked inspiratory dyspnea because of collapse of the nasal planum on inhalation, secondary to destruction of the underlying bony structures
