# Supplementary material for: Nasal phaeohyphomycosis in a cat caused by Cladophialophora boppii
Source: J Vet Intern Med. 2026 Oct 3;40(5):aalag235. doi: 10.1093/jvimsj/aalag235 (PMC13633654; doi:10.1093/jvimsj/aalag235)
Supplement: Supplementary file 1 [file supplementary_material_aalag235.zip › Supplementary Material _ cytology and histopathology.docx]

**CYTOLOGY OF THE NASAL LESION**

Cytology revealed a highly cellular sample in a faintly eosinophilic background containing numerous red blood cells. There were large numbers of long (approximately 50–200 µm), dark-blue, septate, non-branching fungal hyphae with straight, parallel walls measuring approximately the width of a single red blood cell. Smaller numbers of ovoid fungal yeast-like elements were also present; these were usually negatively stained and mildly larger than a red blood cell. The inflammatory population was marked and mixed, consisting predominantly of neutrophils (non-septic) and epithelioid macrophages. Both cell types frequently contained numerous fungal hyphal fragments and yeast-like elements. (Figure 3).

**HISTOPATHOLOGY OF THE NASAL LESION**

Histopathology revealed expansion of the superficial to mid dermis by multiple coalescing granulomas composed of numerous large foamy macrophages, multinucleated giant cells, and neutrophils. These inflammatory cells were associated with abundant fungal yeast and hyphae. The hyphae were septate with non-dichotomous branching and nonparallel walls and were rarely pigmented brown/yellow. Occasionally the yeast formed chains (pseudohypahe). No additional histopathologic features were observed. Overall, the findings were diagnostic of severe granulomatous dermatitis with intralesional pigmented fungal yeast and hyphal forms, consistent with cutaneous phaeohyphomycosis (Figure 4).
