# Supplementary material for: Nasal phaeohyphomycosis in a cat caused by Cladophialophora boppii
Source: J Vet Intern Med. 2026 Oct 3;40(5):aalag235. doi: 10.1093/jvimsj/aalag235 (PMC13633654; doi:10.1093/jvimsj/aalag235)
Supplement: Supplementary file 1 [file supplementary_material_aalag235.zip › Supplementary material_database.docx]

Supplementary material

The 561 ITS nucleotide sequence, derived by forward and reverse read alignment was used to query NCBI BLAST (<https://blast.ncbi.nlm.nih.gov/Blast.cgi>) and the Westerdijk Fungal Biodiversity Institute database using the pairwise alignment tool ([https://wi.knaw.nl/Pairwise alignment](https://wi.knaw.nl/Pairwise%20alignment)). The top unanimous match agreed between both databases: 24MY6808AG matched to the *Cladophialophora boppii* strain CBS 127448 with a score of 1037, 100% query cover and 100% identity using BLAST and 890.75, 100% query cover and 100% identity, using the Westerdijk pairwise alignment tool. Moreover, the results of querying NCBI BLAST using the sequences from type material filter matched the *C. boppii* type strain CBS 126.86, confirming the identity of 24MY6808AG as *C. boppii* (score 987, 96% query cover and 99.45% identity).
